# Dedifferentiated Central Chondrosarcoma: A Clinical, Histopathological, and Immunohistochemical Analysis of 57 Cases

**DOI:** 10.3389/fmed.2021.746909

**Published:** 2021-09-23

**Authors:** Li-Hua Gong, Yong-Bin Su, Wen Zhang, Wei-Feng Liu, Rong-Fang Dong, Xiao-Qi Sun, Ming Zhang, Yi Ding

**Affiliations:** ^1^Department of Pathology, Beijing Jishuitan Hospital, The Fourth Medical College of Peking University, Beijing, China; ^2^Department of Radiology, Beijing Jishuitan Hospital, The Fourth Medical College of Peking University, Beijing, China; ^3^Department of Orthopedic Oncology Surgery, Beijing Jishuitan Hospital, Fourth Medical College of Peking University, Beijing, China

**Keywords:** dedifferentiated, chondrosarcoma, central, imaging, immunohistochemistry

## Abstract

Dedifferentiated central chondrosarcoma (DCCS) is a rare cartilage tumor with invasive biological behavior and a poor prognosis. To better understand the morphological characteristics of this type of tumor and its internal mechanism of dedifferentiation, we retrospectively analyzed 57 cases of DCCS. A total of 29 female and 28 male patients were included, ranging in age from 20 to 76 years, with a median age of 54 years. Fifty-seven cases of DCCS occurred in the pelvis (*n* = 29), femur (*n* = 17), scapula (*n* = 4), tibia (*n* = 2), humerus (*n* = 2), metatarsals (*n* = 1), fibula (*n* = 1), and radius (*n* = 1). Radiologically, DCCS had two different appearances on imaging, with an area showing calcifications of the cartilage forming the tumor juxtaposed to a lytic area with a highly aggressive, non-cartilaginous component. Histopathologically, the distinctive morphological features consisted of two kinds of defined components: a well-differentiated cartilaginous tumor and non-cartilaginous sarcoma. The cartilaginous components included grade 1 (*n* = 38; 66.7%) and grade 2 (*n* = 19; 33.3%) cartilage. The sarcoma components included those of osteosarcoma (*n* = 29; 50.9%), undifferentiated pleomorphic sarcoma (*n* = 20; 35.1%), rhabdomyosarcoma (*n* = 3; 5.2%), fibrosarcoma (*n* = 2; 3.5%), spindle cell sarcoma (*n* = 2; 3.5%) and angiosarcoma (*n* = 1; 1.8%). Immunohistochemistry showed that the expression of p53 and RB in the sarcoma components was significantly higher than that in the cartilaginous components, suggesting that these factors play roles in the dedifferentiation process of chondrosarcoma. DCCS is a highly malignant tumor with a poor prognosis. Except for the patients who were lost to follow-up, most of our patients died.

## Introduction

Dedifferentiated chondrosarcoma (DCS) is a high-grade chondrosarcoma with the bimorphic histological appearance of a conventional chondrosarcoma with abrupt transition to non-cartilaginous sarcoma (1). In the literature, the reported incidence of DCS in chondrosarcoma cases is 10–15% (2). This type of tumor usually occurs between the ages of 50 and 60 years and occurs more frequently in males (3). It is most often located in the pelvis and long bones such as the proximal femur or humerus, the distal femur and the tibia. Dedifferentiation usually originates from either an enchondroma or a low-grade chondrosarcoma (dedifferentiated central chondrosarcoma, DCCS), but it can also originate from a low-grade peripheral chondrosarcoma secondary to a pre-existing osteochondrosarcoma or a solitary osteochondroma (dedifferentiated peripheral chondrosarcoma) (4). Histopathologically, the distinctive morphological features include two kinds of defined components, a well-differentiated cartilage tumor juxtaposed to a high-grade non-cartilaginous sarcoma, and the transition between the two is abrupt. The dedifferentiated components may be conventional osteosarcoma, telangiectatic osteosarcoma (5), undifferentiated pleomorphic sarcoma (UPS), or fibrosarcoma (6). Other rare histological subtypes of the differentiated components may include leiomyosarcoma (7), rhabdomyosarcoma (8), giant cell tumor-like (9–12), gastrointestinal stromal tumor (GIST)-like (13), or epithelial differentiation (14). To better understand the characteristics and dedifferentiation transformation mechanism underlying DCCS, we conducted a retrospective study to analyze the clinicopathological features of 57 patients with DCCS. Additionally, we carried out immunohistochemistry to explore the intrinsic mechanism involved in the process of dedifferentiation in these tumors.

## Methods

### Patients and Surgical Specimens

With approval from the institutional ethics committee and following the research protocol, 57 DCCS cases were retrieved from surgical pathological records between January 2009 and December 2020 at the Department of Pathology, Beijing Jishuitan Hospital. All tissues were fixed in neutral buffered formalin and processed routinely *via* paraffin embedding, and then the sections were prepared and stained with hematoxylin and eosin (HE). Histopathological assessment was carried out according to the WHO Classification of Tumors of Soft Tissue and Bone and reviewed by three pathologists, while clinical and imaging information was obtained from online medical records and surgeons. All cases were treated by surgery, and 28 cases were treated with chemotherapy after surgery.

### Imaging

Pre-operative imaging studies included plain radiographs, computed tomography (CT) and magnetic resonance imaging (MRI). As a routine examination protocol in our hospital, patients with bone tumors are assessed by contrast-enhanced CT or contrast-enhanced MRI. Fifty-one patients underwent plain radiographs with at least two positions; 45 patients were evaluated with CT scans, and 38 patients were assessed by MRI. The images were reviewed on our PACS (Picture Archiving and Communication Systems) by two experienced radiologists (YS, WL).

### Tissue Samples and Immunohistochemistry

Formalin-fixed, paraffin-embedded specimens of DCCS were available for immunohistochemical analysis. Immunohistochemical staining was performed with an automated immunostainer (Autostainer 720, Labvision, San Diego, CA) according to standard heat-induced epitope retrieval and the avidin-biotin-peroxidase complex method. The following cytophenotypic markers were detected: desmin, S-100, p16, RB, p53, Cyclin D1, CDK4, H3k27me3, and Ki-67 (Table 1). Simultaneously, appropriate positive and negative control sections were used. Positive immunostaining was characterized by brown nuclear or cytoplasmic staining under a microscope. Cytoplasmic staining was considered positive for desmin, and nuclear staining was considered positive for S-100, p16, RB, p53, Cyclin D1, CDK4, H3k27me3, and Ki-67. All slides were evaluated independently by two pathologists who were not provided clinical information. The grade of immunoreactivity was defined as follows: negative (**–**); focal positive (+): fewer than 75% of tumor cells were positive; and diffuse positive (++): more than 75% of tumor cells were positive. The Ki67 proliferation index was defined 25% as the threshold value. Agreement was reached by careful discussion when the opinions of the two pathologists (LG, YD) were different.

**Table 1:** Antibodies used for immunohistochemical staining.

**Antigen**	**Antibody**	**Source**	**Type**	**Dilution**
Desmin	ZC18	Zymed	Monoclonal antibody	Prediluted
Myogenin	F5D	DAKO	Monoclonal antibody	1:200
RB	1FB	Zhongshan	Monoclonal antibody	Prediluted
CDK4	EP180	Zhongshan	Polyclonal antibody	Prediluted
Cyclin D1	SP4	Roch	Polyclonal antibody	Prediluted
p53	D0-7	Leica	Monoclonal antibody	1:100
p16	G175-405	Zymed	Monoclonal antibody	Prediluted
S-100	None	Leica	Polyclonal antibody	1:400
H3k27me3	None	Zhongshan	Polyclonal antibody	Prediluted
Ki-67	MM1	Leica	Monoclonal antibody	Prediluted

## Results

### Clinical Characteristics

The patients included 29 females and 28 males, ranging in age from 20 to 76 years, with a median age of 54 years. Fifty-seven cases of DCCS occurred in the pelvis (*n* = 29), femur (*n* = 17), scapula (*n* = 4), tibia (*n* = 2), humerus (*n* = 2), metatarsals (*n* = 1), fibula (*n* = 1), and radius (*n* = 1). In our study, 12 cases (21.1%) were secondary DCCS occurring after central low-grade chondrosarcoma recurrence. The other cases involved primary dedifferentiation (Table 2).

**Table 2:** Clinical summary of DCCS.

**Age**	***n* (%)**
≥50 years	34 (59.7)
<50 years	23 (40.3)
**Sex**	
Female	29 (50.9)
Male	28 (49.1)
**Location**	
Pelvis	29 (50.8)
Femur	17 (29.8)
Scapule	4 (7.0)
Tibia	2 (3.5)
Humerus	2 (3.5)
Fibula	1 (1.8)
Radius	1 (1.8)
Metatarsals	1 (1.8)
**Classification**	
Primary	45 (78.9)
Secondary	12 (21.1)
**Grade of chondrogenic component**	
G1	38 (66.7)
G2	19 (33.3)
**Type of dedifferentiation**	
Osteosarcoma	29 (50.9)
UPS	20 (35.1)
Rhabdomyosarcoma	3 (5.2)
Fibrosarcoma	2 (3.5)
Spindle cell sarcoma	2 (3.5)
Angiosarcoma	1 (1.8)
**Prognosis**	
Disease-free survival	27 (47.4)
Dead	23 (40.3)
Missed follow-up	7 (12.3)

*G1, grade 1; G2, grade 2; UPS, undifferentiated pleomorphic sarcoma*.

### Imaging Characteristics

**Figure 1:**
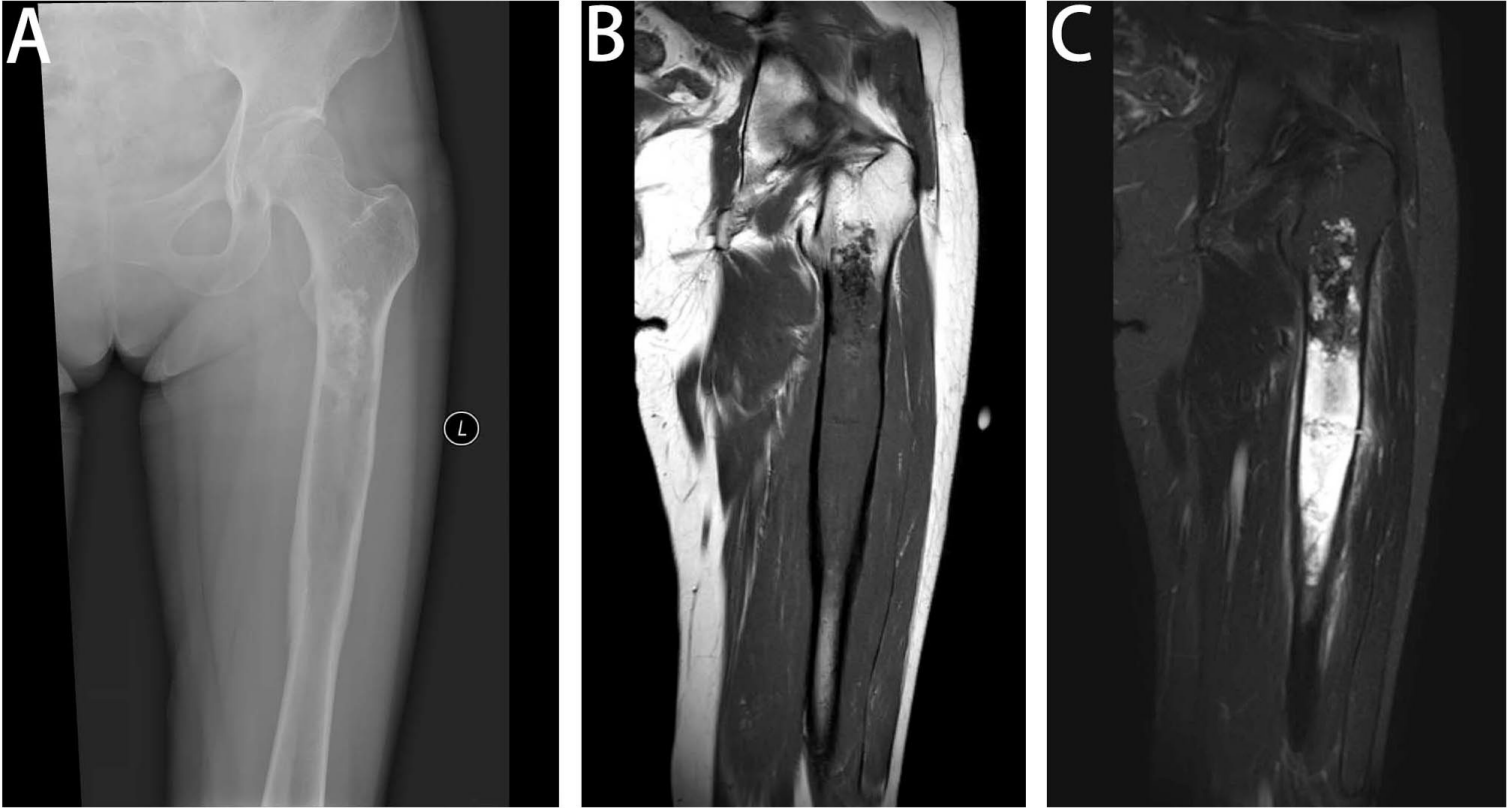
Radiologic features of DCCS. **(A)** Anteroposterior view of the left femur. Radiographs show a large lytic lesion of the femur, with cortical remodeling and a poorly defined margin of the distal part. The proximal part of the lesion has popcorn-like matrix calcifications. **(B)** Coronal T1-weighted MR image of the left femur. MR imaging shows a large destruction in the medullary cavity of the femur, with low signal on the T1-weighted image. **(C)** Coronal fat-suppressed T2-weighted MR image of the left femur. On fat-saturated T2-weighted images, the existence of the two components is recognized. The proximal third of the lesion has areas of low signal representing matrix mineralization and areas of high signal representing the high water content of the cartilage matrix, but the distal two-thirds has a heterogeneous predominantly high signal and peritumoral edema, showing bimorphism of the lesion.

**Figure 2:**
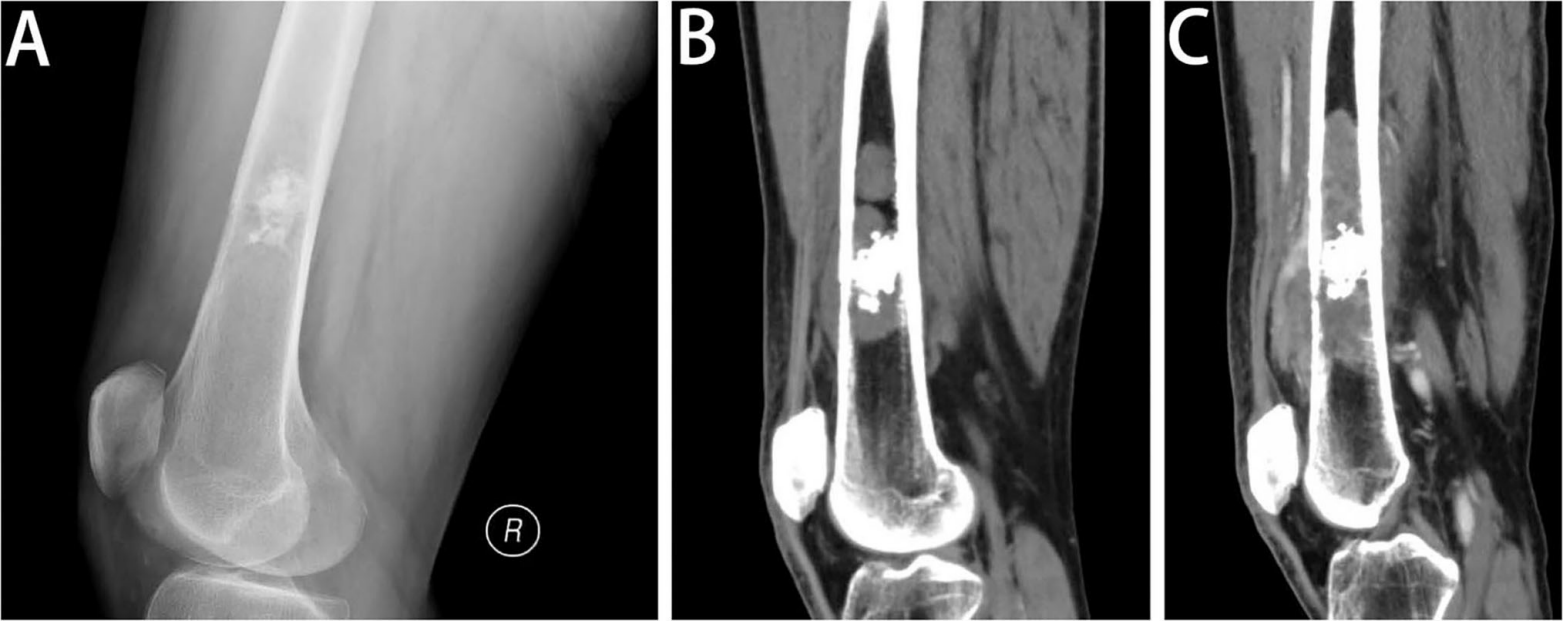
Radiologic features of DCCS. **(A)** Lateral view of the left femur. Radiography of the distal femur shows a lesion in the medullary cavity with calcifications, with subtle destruction of the anterior cortex and anterior soft tissue mass. **(B)** Sagittal CT scan of the left femur in the soft tissue window. The sagittal CT images demonstrate that the lesion is multifocal, with penetration of the cortex and considerable soft tissue components. In addition to the calcific foci, the lesion contains more areas of lytic destructive changes. **(C)** Sagittal post-contrast CT scan of the left femur in the soft tissue window. One and a half months later, the sagittal post-contrast CT image in the soft tissue window shows the lesion expanding into a large area of bone destruction, with heterogeneous enhancement.

Most of the tumors were located in the pelvis and femur. Lesions often exhibit ill-defined intramedullary destruction with calcific foci and associated cortical permeation and soft tissue masses and with heterogeneous contrast enhancement on imaging. The most characteristic features of images consist of two different appearances on imaging, with an area showing calcifications or hyperintense chondral component of the cartilage-forming tumor juxtaposed to a lytic area involving a highly aggressive, non-cartilaginous component with a soft tissue mass, which frequently reflects the presence of undifferentiated pleomorphic sarcoma or osteosarcoma (Figures 1, 2). A total of 21.6% (11/51), 75.6% (34/45), and 50% (19/38) of lesions showed this biphasic pattern on plain radiograph, CT and MRI, respectively. This bimorphic pattern was characteristic and was appreciated more clearly on CT images than on radiography.

### Gross Pathological Features

**Figure 3:**
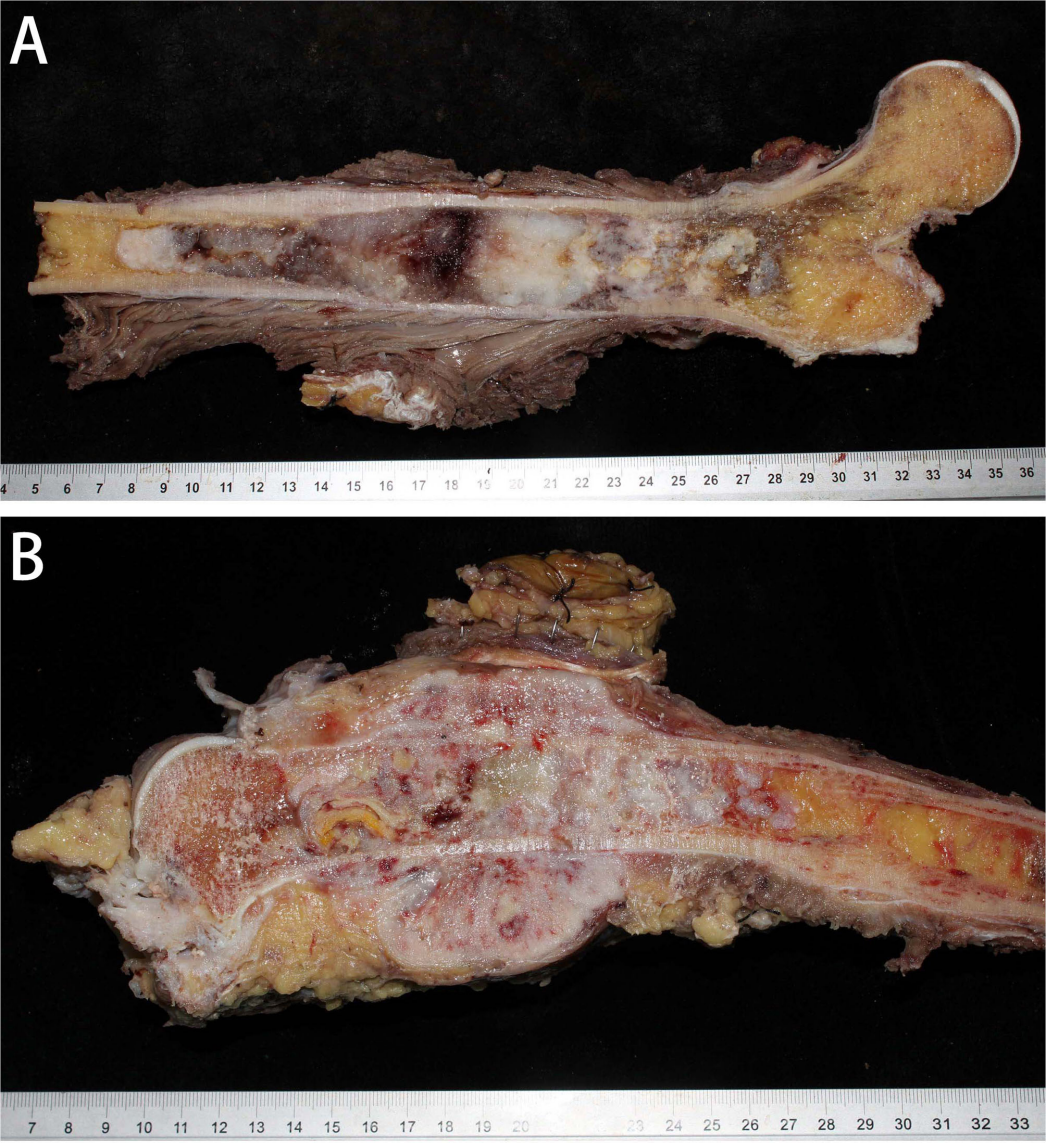
Gross specimens of DCCS. **(A)** The tumor was located in the medullary cavity and was composed of porcelain white cartilage and red fish-like tissue. **(B)** The tumor in the marrow cavity was cartilage-like with invasion of the surrounding soft tissues, forming an osteosarcoma structure.

Grossly, the tumor was located in the medullary cavity. The cartilaginous and DCS components are distinct. The cartilaginous component was blue-gray, translucent and fragile, whereas the dedifferentiated components were fresh, pale, soft, tough or hard (Figure 3A). The sarcoma components could invade the cortex of bone, forming a soft tissue mass (Figure 3B).

### Histopathological Features

**Figure 4:**
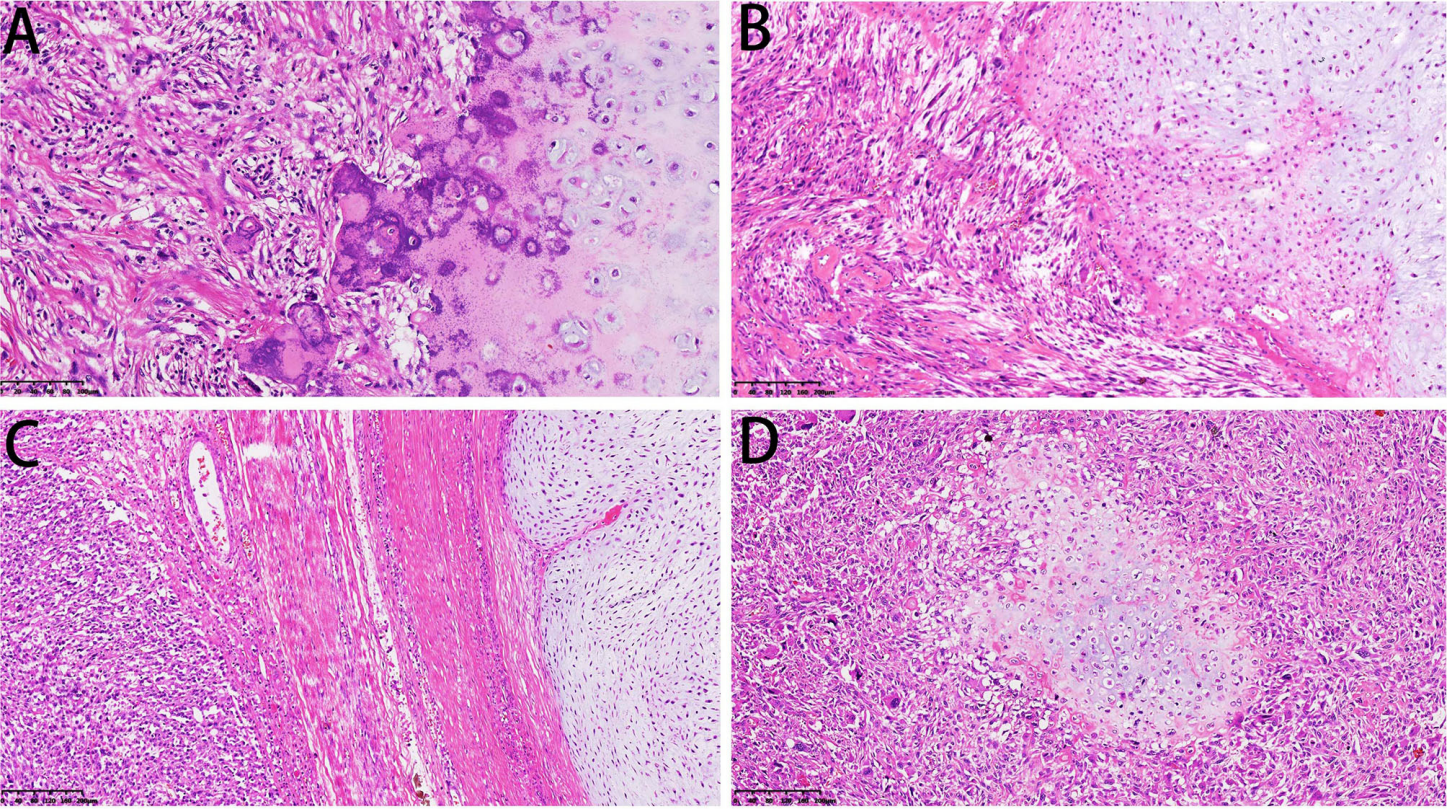
Microscopical features of DCCS. **(A)** The cartilage component shows a grade I cartilage structure, and the sarcoma component shows a low-grade sarcoma morphology (HE, 200×). **(B)** The cartilage component shows a grade II cartilage structure, and the sarcoma component shows the morphology of myxofibrosarcoma (HE, 200×). **(C)** Thick fibrous separation was located between the cartilage component and the sarcoma component (HE, 100×). **(D)** Well-differentiated cartilage nodules surrounded by sarcoma components (HE, 100×).

Microscopically, the DCCS cases showed a typical chondrosarcoma structure and non-chondrogenic sarcoma structure. The types of chondrosarcoma include chondrosarcoma grade I and chondrosarcoma grade II. Grade I cartilage may mimic normal hyaline cartilage. In 38/57 cases, the cartilaginous component consisted of chondrosarcoma grade I, which is weakly to moderately cellular and hyperchromatic, with no mitoses (Figure 4A). Nineteen cases of chondrosarcoma grade II were found. Chondrosarcoma grade II is more cellular, with a greater degree of nuclear atypia, and mitoses can be found (Figure 4B). The perilobular and interlobular cells of chondrosarcoma grade II are rich and pleomorphic. Myxoid changes were found in 22 cases, primarily in chondrosarcoma grade II components.

The dedifferentiated components usually transition abruptly to distinct cartilaginous components. We observed an obvious line of demarcation as described in the WHO classification. Fiber bundles were present in some cases and absent in others (Figure 4C). Additionally, in some cases, sarcoma could be the major component, and the focal cartilaginous component was located in it, forming an island structure (Figure 4D). The dedifferentiated components showed multiple features, including those of high-grade sarcomas, namely, osteosarcoma (*n* = 29), UPS (*n* = 20), rhabdomyosarcoma (*n* = 3), fibrosarcoma (*n* = 2, the diagnostic criteria refer to adult fibrosarcoma in the WHO classification) and angiosarcoma (*n* = 1). Additionally, there were low-grade components, such as grade I or II spindle cell sarcoma (*n* = 2) (Table 1).

**Figure 5:**
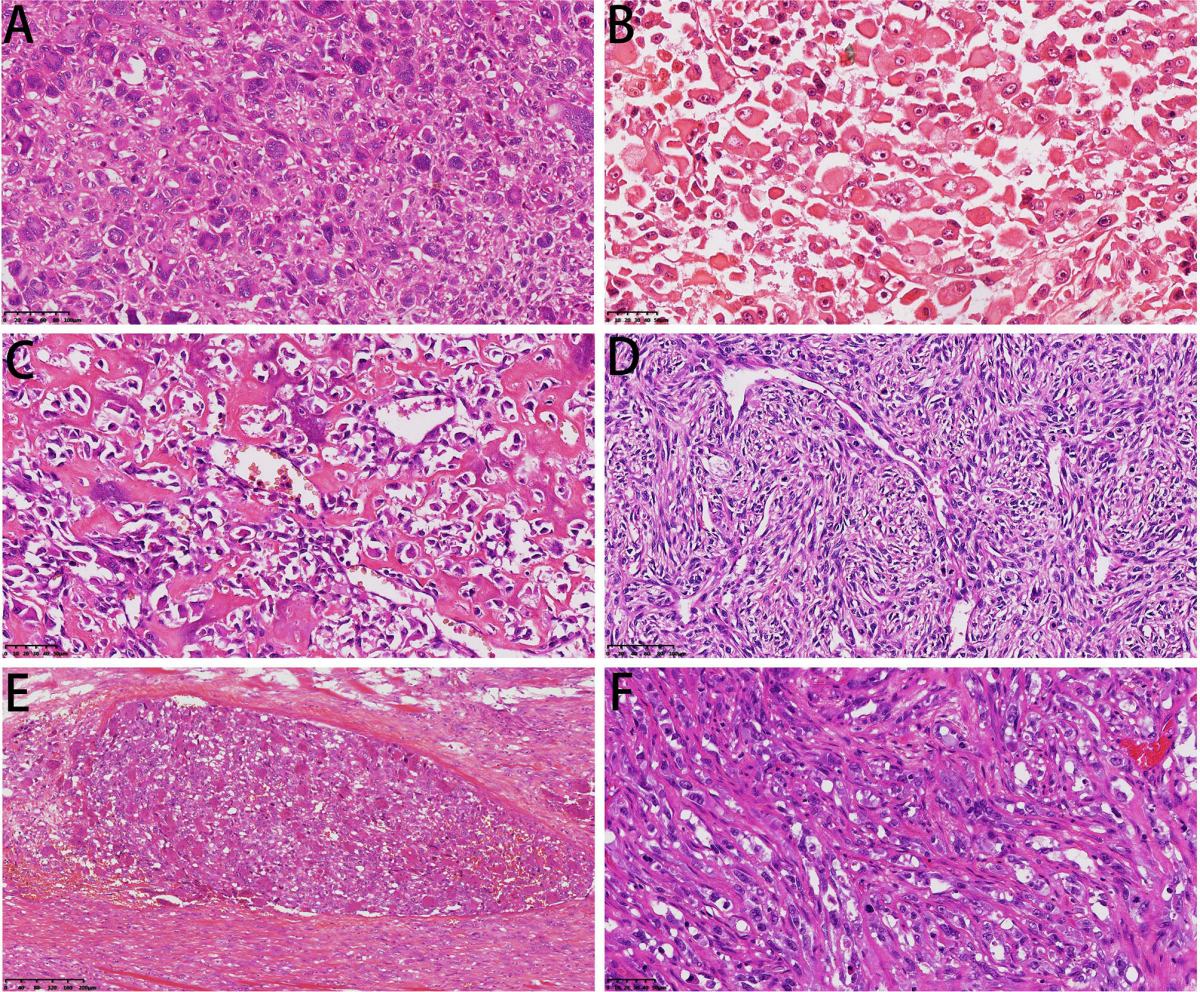
Microscopical features of DCCS. **(A)** Diffuse multinuclear tumor giant cells were dispersed in sarcoma components (HE, 200×). **(B)** The tumor cells showed characteristics of striated muscle differentiation with enriched cytoplasm (HE, 300×). **(C)** The sarcoma component was conventional osteosarcoma (HE, 300×). **(D)** Osteosarcoma tissue showed perivascular arrangement (HE, 200×). **(E)** Intravascular emboli showed a giant cell-rich structure (HE, 100×). **(F)** The sarcoma component showed epithelioid angiosarcoma differentiation (HE, 300×).

In the UPS, sarcoma showed high-grade cell pleomorphism and atypia, some zones with bizarre multinuclear tumorous giant cells (Figure 5A), and some zones with epithelioid morphology. The high-grade myxoid zone also existed, forming high-grade myxoid fibrosarcoma features. In two cases, pleomorphic rhabdomyosarcoma differentiation occurred. The cells were large, round or polygonal with abundant pink cytoplasm and unusual nuclei, similar to rhabdomyosarcoma (Figure 5B). In one case, the tumor invaded the lymph node. Only dedifferentiated components could be found in the lymph node without cartilaginous components.

In the osteosarcoma component, the cells were highly anaplastic and pleomorphic with enlarged and darkly stained nuclei (Figure 5C). A focal hemangiopericytoma-like pattern was also found (Figure 5D). The important and necessary characteristics for the diagnosis of osteosarcoma are the production of osteoids by malignant tumor cells. Osteoids were dense, pink, and amorphous intercellular material with or without calcification. Osteosarcoma has a broad morphological spectrum. Small cell osteosarcoma had uniform small cells with scant cytoplasm, with little pink osteoid production. The nuclei were round to oval, and the chromatin was fine. The osteoclast-type giant cells scattered in the tumor cells formed the giant cell-enriched variant of osteosarcoma in one case. Additionally, in this case, blood vessel invasion could be found (Figure 5E). Two cases showed focal telangiectatic osteosarcoma features. The tumor was composed of cystic spaces simulating an aneurysmal bone cyst, but pleomorphic tumor cells with focal osteoid formation were scattered in the septa-like structures.

**Figure 6:**
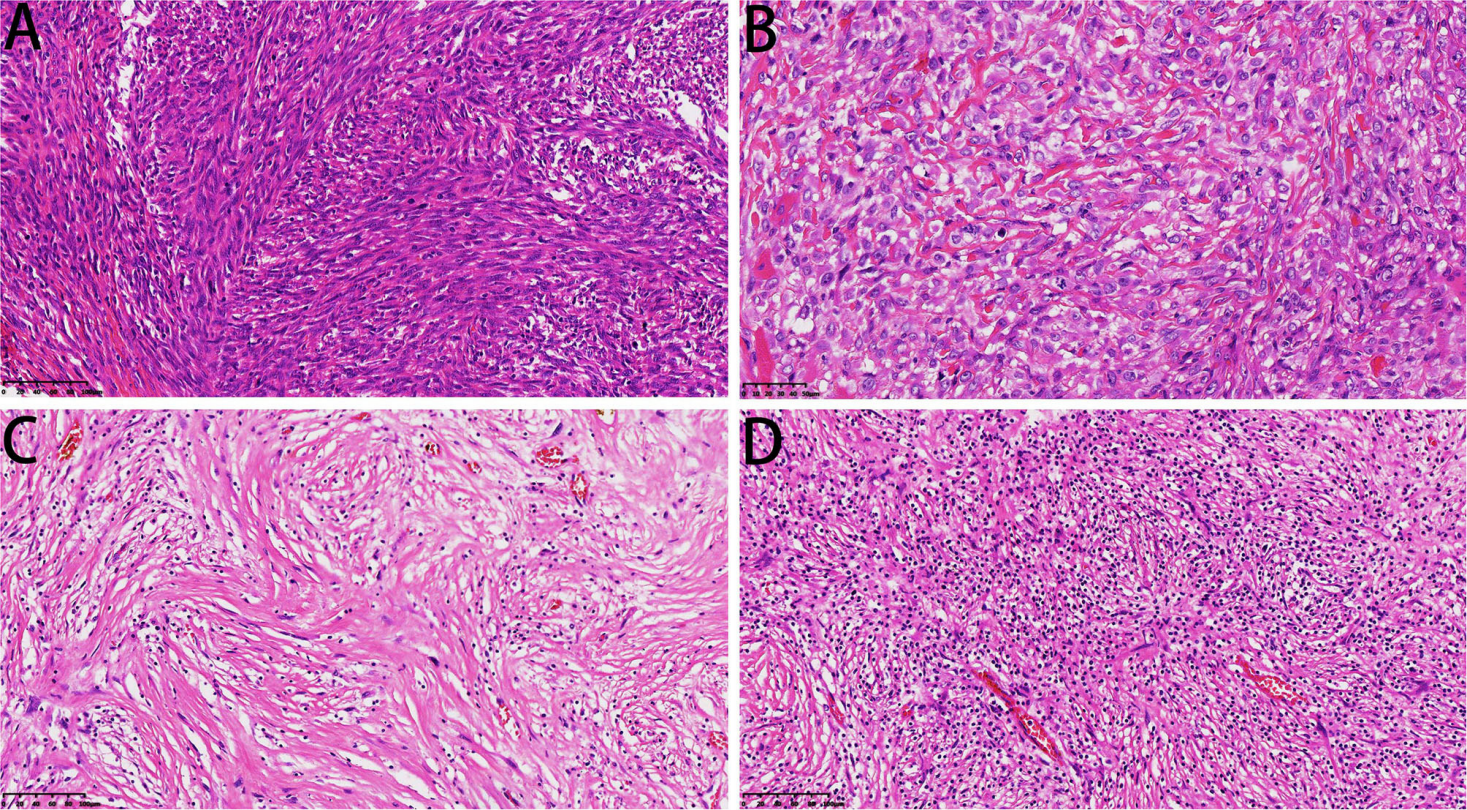
Microscopic features of DCCS. **(A)** The spindle cells were arranged in a bundle-like fibrosarcoma (HE, 200×). **(B)** The sarcoma component resembled sclerotic epithelioid fibrosarcoma (HE, 300×). **(C)** Low-grade sarcoma components showed storiform structures (HE, 200×). **(D)** Low-grade areas showed inflammatory fibroblastoma morphology (HE, 200×).

One case showed epithelioid angiosarcoma features (Figure 5F). The fibrosarcoma was composed of relatively monomorphic spindle cells with a “herringbone” growth pattern and sometimes also formed a synovial sarcoma-like structure (Figure 6A). In some areas, the tumor cells were round and epithelioid with interstitial fiber formation, similar to sclerosing epithelioid fibrosarcoma features (Figure 6B). Two cases showed low-grade spindle cell sarcoma, with mild-moderate atypia, and some areas had storiform patterns, forming a benign fibrohistiocytoma structure (Figure 6C); some areas had a distinctive inflammatory infiltrate with aggregates of plasma cells and lymphocytes mimicking the inflammatory myofibroblastic tumor (Figure 6D).

### Immunohistochemical Features

**Figure 7:**
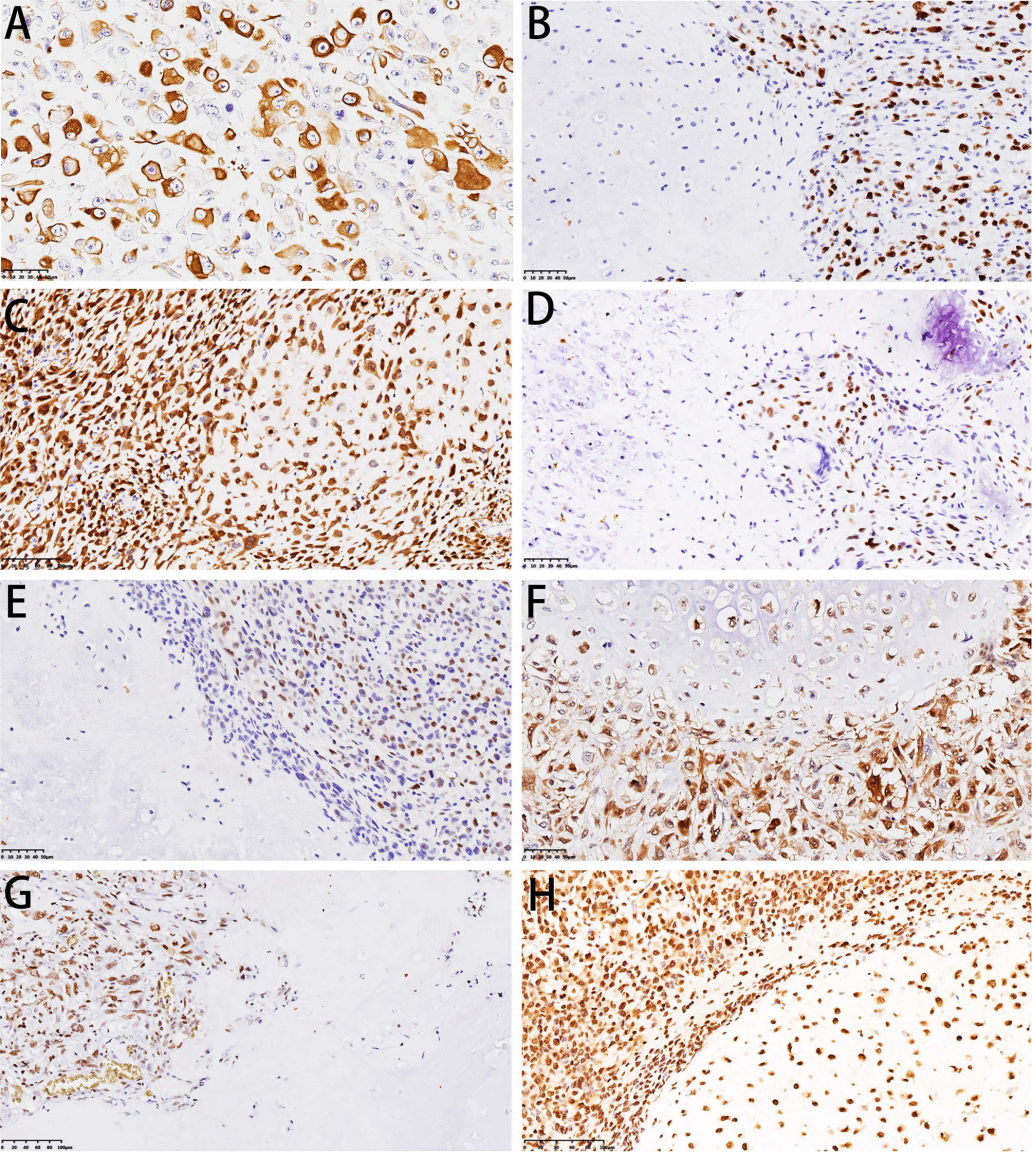
Immunohistochemical features of DCCS. **(A)** Immunohistochemistry showed desmin positivity for striated muscle differentiation. **(B)** Immunohistochemistry showed that Ki-67 was highly expressed in sarcomas. **(C)** Immunohistochemistry showed dense expression of p16 in cartilage and sarcomas. **(D)** Immunohistochemistry showed p53 expression in sarcomas. **(E)** Immunohistochemistry showed Cyclin D1 expression in sarcomas. **(F)** Immunohistochemistry showed that both cartilage and sarcoma components expressed CDK4. **(G)** Immunohistochemistry showed RB expression in sarcoma. **(H)** Immunohistochemistry showed that H3K27me3 was expressed in both the cartilage and sarcoma components.

Immunohistochemical staining showed strong positivity for desmin in the rhabdomyosarcoma differentiation components in two cases (Figure 7A). The Ki-67 index revealed active cell proliferation in the sarcoma components; 79.3% cases showed a Ki-67 value >25%, with only individual cells in the cartilage portion showing proliferative activity, and the Ki-67 value was <25% in all cartilage portions (Figure 7B). Staining for p16 was negative in sarcoma and original cartilaginous lesions in most cases; only in some cases were both components positive, and the sarcoma component showed stronger and more diffuse staining than the cartilaginous components (Figure 7C). Staining for p53 was diffusely positive in the nuclei of tumor cells in the dedifferentiated components of 54.4% cases (Figure 7D) but almost undetectable in the chondrosarcoma component, except for one case with grade II cartilage. The expression pattern of Cyclin D1 was similar to that of p16. In 29.9% of cases, the sarcoma was focal positive, while in only 5.3% of cases, the cartilage was positive. The expression intensity of the sarcoma portion was much higher than that of the cartilage portion (Figure 7E). In some cases, although CDK4 was positive in both the cartilage and sarcoma portions, the cartilage portion showed focal positive expression, while the sarcoma portions showed mainly diffuse expression (Figure 7F). RB was not expressed in the cartilage portion of all cases, but it showed positivity (73.7%) in the sarcoma portion (Figure 7G). H3k27me3 was diffuse positive in the cartilage in all cases, and the sarcoma components were diffuse positive in 89.5% of cases; in only six cases, H3k27me3 showed focal expression (Figure 7H). The results of immunohistochemical staining are depicted in Table 3.

**Table 3:** Immunohistochemical results of DCCS.

	**Negative (–)** ***n*** **(%)**		**Focal positive (+)** ***n*** **(%)**		**Diffuse positive (++)** ***n*** **(%)**	
	**Chondrogenic**	**Sarcoma**	**Chondrogenic**	**Sarcoma**	**Chondrogenic**	**Sarcoma**
Desmin	57 (100)	54 (94.7)	0	3 (5.3)	0	0
Myogenin	57 (100)	54 (94.7)	0	3 (5.3)	0	0
S-100	0	57 (100)	0	0	57 (100)	0
RB	57 (100)	15 (26.3)	0	23 (40.4)	0	19 (33.3)
CDK4	45 (78.9)	29 (50.9)	12 (21.1)	20 (35.1)	0	8 (14.0)
Cyclin D1	54 (94.7)	40 (70.2)	3 (5.3)	14 (24.6)	0	3 (5.3)
p53	56 (98.2)	31 (54.4)	1 (1.7)	19 (33.3)	0	7 (12.3)
p16	47 (82.4)	45 (78.9)	4 (7.0)	6 (10.5)	6 (10.5)	6 (10.5)
H3k27me3	0	0	0	6 (10.5)	57 (100)	51 (89.5)

## Prognosis

The patients were followed up from 2 to 127 months. The median follow-up time was 59 months. Seven patients were lost to follow-up. Twenty-three patients died of DCCS. The remaining 27 patients showed no evidence of recurrence and survived disease free.

## Discussion

In 1971, Dalin and Beabout first fully described the dedifferentiation of low-grade chondrosarcomas, which is rare, and reported a combination of well-differentiated chondrosarcoma of the ordinary type and juxtaposed zones of anaplastic fibrosarcoma or osteogenic sarcoma. The incidence of peripheral DCS has ranged between 8.9 and 13.7% of all DCS cases (15). Staals et al. (16) found 109 central DCS cases out of 784 central chondrosarcomas (13.9%). In our hospital, from 2009 to 2019, 694 cases of chondrosarcoma were treated at our institution. Among them, 57 patients were diagnosed with DCCS, which accounts for an 8.2% dedifferentiation rate and does not include peripheral DCS. DCCS usually affects the long bones, such as the proximal femur, humerus and tibia. The femur was involved twice as often as the pelvis in DCCS, and there were slightly more males than females at the Razzoli Institute (17). However, in our series, the pelvis was a more common location than the femur, and there were more females than males. These differences may be explained by the selection bias associated with more complicated lesions at the Orthopedic Oncologic Center.

The biphasic pattern is the most characteristic feature of imaging and is appreciated more clearly on contrast-enhanced CT images and MRI than on radiography. Plain radiography has a poor ability to assess the soft tissue mass of the aggressive part. MRI has been investigated well in DCCS and proved to be a useful imaging modality. On MR fluid-sensitive sequences such as T2-weighted or STIR images, chondral tumors are hyperintense, whereas dedifferentiated tumors have reduced signal intensity or heterogeneous signal intensity. Based on our results, CT is a good imaging technique to show matrix calcifications of the underlying chondral tumor and osteoid production by osteosarcoma, and with post-contrast images, the soft tissue mass of dedifferentiated tumors is clearly demonstrated with avid enhancement. Imaging findings of the biphasic pattern are important to help pathologists choose the appropriate areas to observe.

DCCS can take place at the first surgery for endochondroma or low-grade chondrosarcoma, which is called synchronous (primary) DCCS, and can also be found at recurrence after resection of these chondrogenic tumors, which is called metachronous (secondary) DCCS. When recurrence occurs at the same site of the chondrosarcoma, the lesion may include the chondrosarcoma structure, which must be combined with the previous pathology to make a precise diagnosis. In addition, the dedifferentiated component may be very small; therefore, pathologists should sample cartilaginous tumors thoroughly, paying special attention to zones that seem grossly abnormal. For the diagnosis of primary DCCS, due to limitations of biopsy, it is often difficult to obtain both cartilage and dedifferentiated components at the same time, which leads to errors in diagnosis. CT-guided puncture could greatly improve the accuracy of diagnosis. For the diagnosis of secondary DCCS, biopsy combined with history could be easier to diagnose.

The Evans grading system classifies chondrosarcoma into grades I, II, and III (low, intermediate and high grade, respectively) according to nuclear morphology, mitotic activity and the degree of cellularity (18). Similar to reports in the literature, the cartilage components in our case had grade I and II morphology, and no grade III morphology was observed. It should be noted that cells around the grade II cartilage lobules are often densely distributed and should be distinguished from grade III. Moreover, in cases of secondary dedifferentiation, the grade of cartilage components are higher than that in the original case. Myxoid degeneration of cartilage was also common, but it was not a criterion for grading. The dedifferentiation components may show multiple features, and UPS, fibrosarcoma and osteosarcoma were the main types. Rare types, such as small cell osteosarcoma, could also be seen. In many cases of osteosarcoma, osteoid production may occupy a small part and show multiple patterns. We also observed other rare types of histology, including angiosarcoma and rhabdomyosarcoma. Similar to Wick's description (19), in our cases, the dedifferentiated component also showed low-grade spindle cell tumor features, which existed in the form of a storiform structure, with focal lymphocyte infiltration, generating an inflammatory myofibroblastoma-like morphology. Consistent with literature reports of giant cell tumor-like morphology, there were indeed a large number of giant cells in our cases, in either giant cell-rich undifferentiated sarcomas or giant cell-rich osteosarcomas, especially in the latter, where the focal lesion may present low-grade features similar to those of giant cell tumors of bone. These giant cells were all benign and behaved differently from tumor giant cells in undifferentiated pleomorphic sarcoma.

The origin of the dedifferentiation component remains controversial. Sanerkin and Woods (20) suggested that two completely different tumor cell components developed from multipotent mesenchymal stem cells into different cell clones and then differentiated into different neoplastic cell components (collision tumor). In contrast to this theory, more evidence supports the monoclonal origin theory that genomic instability causes a common primitive mesenchymal cell progenitor, which possesses both the ability to develop into a differentiated (chondrocytic features) and a dedifferentiated cell population (high-grade sarcoma features) (21). The fact that chondrocytes were found to have the potential to differentiate into osteoblastic cells supports this theory. Therefore, chondrocyte transition to other tissues may also be possible. The molecular mechanisms of chondrosarcoma dedifferentiation transition also must be further explored.

Morphological observation shows that there is more active mitosis in the sarcoma component than in the cartilage component. Moreover, the Ki-67 proliferation index was significantly increased in sarcoma components by immunohistochemical staining. Therefore, we performed immunohistochemical analysis of cell cycle-related molecules. p53 plays a role in cell cycle regulation, apoptosis, genomic stability, and inhibition of angiogenesis. Studies have shown that p53 mutation is the main mutant gene in high-grade chondrosarcoma (22, 23) and DCS. In DCS, p53 mutation or loss of heterozygosity (LOH) was detected only in the advanced dedifferentiated components (24). It has been reported that p53 is overexpressed in dedifferentiated areas, while chondrosarcoma areas have only focal weakly positive or negative expression (25). Bovée et al. (26) found that p53 was expressed in both chondrosarcoma and dedifferentiated regions. These differences were thought to be due to the different cartilage grades; grade II cartilage is more likely to be p53 positive than grade I cartilage. According to these findings, in our one case, p53 was weakly positive in grade II cartilage; the other cases were negative in the cartilage area, but p53 was positive to varying degrees in the sarcoma area of 26 cases. Another study confirmed the role of the p53 pathway in the high-grade progression of chondrosarcoma (27). Therefore, we believe that p53 may be related to the malignant transformation of chondrosarcoma dedifferentiation.

The retinoblastoma (RB) protein controls E2F-mediated gene transcription activation and is a key factor for cells entering S phase and cell cycle progression. Loss of RB function is an essential step in tumorigenesis. The LOH of RB is associated with high-grade cartilage tumors and is thought to occur only in the anaplastic component (28, 29). We found that the expression of RB occurs in dedifferentiated components without exception and that the cartilage components are all negative. Therefore, we hypothesized that abnormal expression of RB could induce cartilage tumor stem cells to accelerate cell cycle progression and combine with p53 gene mutation to lead to the development of sarcoma.

Amary et al. (30) showed that p16 copy number variation could be found in high-grade chondrosarcoma (GII, GIII and dedifferentiated) and may be associated with tumor progression. Analysis of a DCS cell line suggests that deletion of the p16 gene plays a major role in the malignant phenotype of DCS (31). p16 regulates the cell cycle by inhibiting CDK4 and Cyclin D1. We found that in most cases, p16 showed a loss of expression in cartilage and dedifferentiated components, which may suggest that the inhibition of CDK4 and Cyclin D1 was reduced, prompting tumor cells to enter the cell cycle progression. Therefore, p16 may also play a role in the high-grade progression of chondrosarcoma.

Our immunohistochemical results showed that positive cells were often located at the junction of cartilage nodules and dedifferentiated components—that is, cells around the cartilage lobules. We believe that the cells surrounding the cartilage lobules are germinal cells or tumor stem cells of cartilage tumors, which could generate new chondrocytes and differentiate in different directions. This expression suggests that these cells terminally differentiate into dedifferentiated sarcoma components. In other words, the cartilage component and the sarcoma components are from the same source. However, the mechanism by which cartilage tumor stem cells differentiate into sarcoma cells remains unknown.

H3K27me3 deficiency was found in 34–75% of malignant peripheral nerve sheath tumors (MPNSTs), and the loss of H3K27me3 expression by immunohistochemical staining could provide a diagnostic clue for MPNSTs (32). The histology of DCS that was deficient in H3K27me3 was different from that of DCS that fully expressed H3K27me3 because the former exhibited characteristics of malignant peripheral nerve sheath tumors (33). In our study, six cases showed a dispersed reduction in H3K27me3 expression, and the morphologies were spindle cell tumors. Nevertheless, the basis for the diagnosis of MPNST is insufficient.

The tumors must be differentiated from other cartilaginous tumors and non-chondroid mesenchymal tumors, which may be confused on clinical and roentgenologic grounds. Occasional high-grade chondrosarcoma, which has dense spindle cells around the periphery of cartilaginous lobules, could be explained as the primitive multipotential stem cell of chondrosarcoma, but dedifferentiation does not occur. In mesenchymal chondrosarcoma, there is also relatively well-differentiated cartilage, but the compact proliferated cells adjacent to the cartilage are composed of round or oval primitive cells, and a perivascular structure may exist. However, in DCCS, the high-grade non-chondroid component showed more variable features. In chondroblastic osteosarcoma, the cartilage is usually grade II or grade III, and there is either a gradual rather than rapid transition between the cartilage and the high-grade osteosarcoma zones or a mixing of the two components. Other non-chondroid mesenchymal tumors, such as UPS or fibrosarcoma of bone, may be mixed with secondary DCS without cartilage areas. Therefore, careful sampling combined with patient history is important.

## Conclusions

We reported histological and immunohistochemical characteristics of a large institutional series of DCCS. Immunohistochemical analysis showed that p53 and RB may be related to malignant transformation of DCCS. Because of its rareness, DCCS should be diagnosed carefully and differentiated from other primary malignant sarcomas. Recognizing DCCS is important because of its aggressive clinical behaviors, frequency of recurrence and poor prognosis.

## Data Availability Statement

The original contributions presented in the study are included in the article/supplementary material, further inquiries can be directed to the corresponding authors.

## Ethics Statement

This study was approved by the Ethic Committee of Beijing JishuiTan Hospital. This retrospective study of formalin-fixed and paraffin-embedded specimen was waived of obtaining patient consent.

## Author Contributions

L-HG and YD conceived the study and designed the experiments. L-HG, WZ, and R-FD performed the experiments. Y-BS and W-FL collected and analyzed the radiology data. X-QS and MZ collected the immunohistochemical data. All authors read and approved the final manuscript.

## Conflict of Interest

The authors declare that the research was conducted in the absence of any commercial or financial relationships that could be construed as a potential conflict of interest.

## Publisher's Note

All claims expressed in this article are solely those of the authors and do not necessarily represent those of their affiliated organizations, or those of the publisher, the editors and the reviewers. Any product that may be evaluated in this article, or claim that may be made by its manufacturer, is not guaranteed or endorsed by the publisher.
